# Long‐term oncological outcomes of follicular thyroid cancer in adolescents and young adults: A nationwide population‐based study

**DOI:** 10.1002/wjs.12279

**Published:** 2024-07-07

**Authors:** Daniël J. van de Berg, Christiaan F. Mooij, A. S. Paul van Trotsenburg, Hanneke M. van Santen, Sheila C. E. J. Terwisscha van Scheltinga, Menno R. Vriens, Schelto Kruijff, Els J. M. Nieveen van Dijkum, Anton F. Engelsman, Joep P. M. Derikx

**Affiliations:** ^1^ Department of Pediatric Surgery Emma Children's Hospital Amsterdam University Medical Centers University of Amsterdam Amsterdam The Netherlands; ^2^ Department of Pediatric Endocrinology Emma Children's Hospital Amsterdam University Medical Centers University of Amsterdam Amsterdam The Netherlands; ^3^ Department of Pediatric Endocrinology Wilhelmina Children's Hospital Utrecht University Medical Center University of Utrecht Utrecht The Netherlands; ^4^ Department of Pediatric Oncology Princess Máxima Center Utrecht The Netherlands; ^5^ Department of Pediatric Surgical Oncology Princess Máxima Center Utrecht University Medical Center University of Utrecht Utrecht The Netherlands; ^6^ Department of Surgery University Medical Center Utrecht University of Utrecht Utrecht The Netherlands; ^7^ Department of Surgery University Medical Center Groningen University of Groningen Groningen The Netherlands; ^8^ Department of Molecular Medicine and Surgery Karolinska Institutet Stockholm Sweden; ^9^ Department of Surgery Amsterdam University Medical Centers University of Amsterdam Amsterdam The Netherlands

**Keywords:** adolescents and young adults, AYAs, follicular thyroid cancer, long‐term oncological outcomes, recurrence, survival

## Abstract

**Background:**

Follicular thyroid carcinoma (FTC) in adolescents and young adults (AYAs) is rare and data on long‐term oncological outcomes are scarce. This study aimed to describe the long‐term recurrence and survival rates of AYAs with FTC, and identify risk factors for recurrence.

**Methods:**

This is a retrospective cohort study combining two national databases, including all patients aged 15–39 years, diagnosed with FTC in The Netherlands between 2000 and 2016. Age, sex, tumor size, focality, positive margins, angioinvasion, pT‐stage, and pN‐stage were included in a Cox proportional hazard model to identify risk factors for recurrence.

**Results:**

We included 192 patients. Median age was 31.0 years (IQR 24.7–36.3) and the male to female ratio was 1:4.1. Most patients presented with a minimally invasive FTC (MI‐FTC) (95%). Five patients presented with synchronous metastases (2.6%), including two with locoregional metastases (1%) and three with distant metastases (1.6%). During a median follow‐up of 12.0 years, three patients developed a recurrence (1.6%), of which one patient developed a local recurrence (33%), and two patients a distant recurrence (67%). Five patients died during follow‐up (2.6%). Cause of death was not captured. A Cox proportional hazard model could not be performed due to the low number of recurrences.

**Conclusions:**

FTC in AYAs is generally characterized as a low‐risk tumor, as it exhibits a very low recurrence rate, a high overall survival, and it typically presents as MI‐FTC without synchronous metastases. These findings underscore the favorable long‐term oncological prognosis of FTC in AYAs.

## 1 INTRODUCTION

The vast majority of thyroid tumors are either papillary thyroid carcinoma (PTC) or follicular thyroid carcinoma (FTC), collectively referred to as differentiated thyroid cancer (DTC).^1^ One of the major prognostic factors of DTC is age at diagnosis, which is incorporated into the tumor, nodes, metastases‐staging (TNM), resulting in a more favorable oncological prognosis for patients younger than 55 years.^2^ Among adolescents and young adults (AYAs; with varying age limits from approximately 15–39 years of age), thyroid cancer now ranks as the third most prevalent cancer.^3^ Additionally, its incidence is rising in this age group.^4^ Still, reports on thyroid cancer in the AYA population are scarce and focus exclusively on PTC. AYA individuals with PTC exhibit large tumors with frequent lymph node involvement at diagnosis, and have a relatively high recurrence rate ranging from 7% to 9%.^5^, ^6^, ^7^ However, they also demonstrate a favorable overall and cause‐specific 5‐year survival rate between 97% and 100%.^5^, ^7^ Yet, to the best of our knowledge, there is a paucity of data concerning the long‐term oncological outcomes of AYAs with FTC. Understanding the unique aspects of FTC in AYAs may constitute the initial step toward more tailored counseling, treatment, and follow‐up care within this particular age group. Therefore, the primary aim of this study is to describe the long‐term recurrence and survival rates of AYAs aged 15–39 years with FTC in a national cohort, and identify risk factors for recurrence. The secondary aim of this study is to describe the tumor characteristics and treatment modalities within this group.

## 2 MATERIALS AND METHODS

This study was conducted as retrospective, nationwide, population‐based cohort study based on the nationwide databases “Netherlands Cancer Registry” (NCR)^8^ and the “Nationwide Network and Registry of Histo‐ and Cytopathology in the Netherlands” (PALGA).^9^ This study is reported according to the Strengthening the Reporting of Observational studies in Epidemiology guidelines,^10^ using the methods in line with the previous published study regarding pediatric PTC and FTC.^11^ This study is approved by the institutional review board of Amsterdam University Medical Centers (registration number #20.319). Nationwide data of patients with FTC between 15 and 39 years of age, in the period from 2000 to 2016, were collected from the NCR.^8^ Patients with oncocytic carcinoma of the thyroid (OCA), follicular variant of PTC, those exhibiting a co‐occurrence of another histological subtype of thyroid cancer alongside FTC, patients whose tumor was discovered by coincidence during autopsy, or patients that were treated previously for another type of tumor were excluded from this study. Recurrence data were collected by matching the cohort with data from PALGA.^9^ Using the database of PALGA and in consultation with a pathologist, all patients were retrospectively reclassified in accordance with the staging criteria of the eighth edition of the TNM classification system.^2^

Primary outcomes included the recurrence and overall survival rate of patients with FTC between 15 and 39 years of age, and to identify risk factors for recurrence. Secondary outcomes included the tumor characteristics (TNM‐stage, diameter of tumor, focality, angioinvasion, and extent of invasion) and treatment modalities. Extent of invasion was categorized into minimally invasive (MI‐FTC) and widely invasive (WI‐FTC), following the original pathology reports from PALGA. For this study, we chose the age cutoff of 15–39 years to define AYAs, following the definition of the National Cancer Institute and the most accepted definitions in the current literature.^12^, ^13^ Recurrence was defined as a cytology‐ or pathology‐proven recurrence after an interval of at least 12 months following primary surgical treatment. Local recurrence was defined as a cytology‐ or pathology‐proven recurrence in one of the cervical lymph node levels of the neck. Distant recurrence was defined as recurrence outside of the cervical lymph node levels of the neck. Last date of follow‐up for survival and recurrence were January 31st, 2021. The following variables were included in the analysis for risk factors for recurrence: age (as continuum), sex, size of the primary tumor, focality, positive surgical margins, pT‐stage, pN‐stage, and angioinvasion. The aforementioned variables were identified in a recent systematic review regarding FTC in patients of all ages.^14^

### 2.1 Statistical analysis

Categorical variables were expressed as numbers and percentages. Continuous variables were expressed as median with interquartile range (IQR) for non‐normally distributed data and as mean with standard deviation for normally distributed data. Missing data were excluded from the analysis. A Cox proportional hazard model was applied on recurrence data to determine independent risk factors. Patients without recurrence were censored at the last follow‐up. Statistical significance was defined using a 2‐sided *a* = 0.05 and/or 95% confidence interval. Statistical analysis was performed using SPSS 28.0 software.

## 3 RESULTS

The initial cohort consisted of 224 patients. In total, 32 patients were excluded based on their pathology diagnosis (OCA, *n* = 23; follicular variant of PTC, *n* = 6; poorly differentiated thyroid carcinoma, *n* = 3). Consequently, 192 patients were included in this study. The median age of diagnosis was 31.0 years (IQR 24.7–36.3); the male to female ratio was 1:4.1.

### 3.1 Long‐term oncological outcomes

Long‐term oncological outcomes are shown in Table 1. Median follow‐up was 12.0 years (IQR 8.0–17.0). During follow‐up, three patients (1.6%) developed a recurrence. Of these, one patient developed a local recurrence 19 months after initial surgery, while two patients developed a distant recurrence at 17 and 23 months after initial surgery, respectively. The patient with a local recurrence was a 39‐year old female who was initially diagnosed with a minimally invasive pT1bNxMx tumor without angioinvasion. She was treated with a two‐staged total thyroidectomy and subsequent radioiodine ablation (RAI). Nineteen months after initial surgery she developed a local recurrence, which was surgically excised. Fifteen months later, she developed another local recurrence. She was treated with left sided lateral lymph node dissection, during which 27 lymph nodes were removed. At that time, no other metastases were found. Yet, 9 months later, multiple metastases were found in level VI, which were excised. At the last date of follow‐up, 12 years after initial surgery, the patient was still alive. Two patients developed a distant recurrence. The first patient, a 39‐year old female presented with a WI pT4aNxM1 tumor with cerebral metastases. She underwent a total thyroidectomy and resection of the cerebral metastases, followed by RAI. Seventeen months after surgery, she developed a distant recurrence in her left ilium, for which an excision was performed. At the last date of follow‐up, 17 years after initial surgery, the patient was still alive. The other patient was a 38‐year old female who was initially diagnosed with a WI pT1bNxM1 with metastases in one of the lumbar vertebrae. She underwent a total thyroidectomy and excision of the lumbar metastases, followed by RAI. Twenty‐three months after the total thyroidectomy, she developed a distant recurrence in one of the right coste. Data on her treatment for this recurrence were not recorded in the database. At the last date of follow‐up, 4 years after initial surgery, this patient had died. The cause of death was not captured in the database.

**TABLE 1:** Long‐term oncological outcomes of 192 AYA's with follicular thyroid carcinoma.

Follow‐up, median in years (IQR)	12.0 (8.0–17.0)
Recurrence, *n* (%)^a^	3 (1.6)
5‐year disease‐free survival, %	98.4
Location, *n* (% of total recurrences)	
Local	1 (33.3)
Distant	2 (66.7)
Consecutive recurrences, *n* (%)	
1	2 (1.0)
3	1 (<1)
Death (to any cause), *n* (%)	5 (2.6)
10‐year overall survival, %	97.4

*Note*: There were no missing variables.

Abbreviations: AYA's, adolescents and young adults; IQR, interquartile range; *n*, number of patients.

^a^ Time to recurrences were 17, 19, and 23 months.

During follow‐up, four other patients also died. All patients were young adults (22, 28, 34, and 36 years old, respectively). The causes of death of these four patients were also not recorded in the database. The 10‐year overall survival was 97.4%.

### 3.2 Risk factors for recurrence

A Cox proportional hazard model to determine risk factors for recurrence could not be performed, as only three recurrences occurred in this cohort.

### 3.3 Tumor characteristics

Tumor characteristics are shown in Table 2. Most patients presented with a pT2 tumor (105 patients; 54.7%). Forty‐eight patients (25.0%) were diagnosed with a pT3a tumor. Four patients (2.1%) presented with extrathyroidal extension invading subcutaneous soft tissue, larynx, trachea, esophagus, or recurrent laryngeal nerve (pT4a). Two patients presented with lymph node metastases (1.0%): a 35‐year old male presented with central lymph node metastases (pN1a) and a 34‐year old female presented with lateral lymph node metastases (pN1b). In 33 patients (17.2%), lymph nodes were either collected or identified in the pathology specimen, but no metastases were found in these lymph nodes (pN0). In 157 patients (81.8%), no lymph nodes were collected (Nx). At presentation, distant metastases were found in three patients (1.5%), of which two patients had skeletal metastases and one had cerebral metastases.

**TABLE 2:** Tumor characteristics of 192 AYA's with follicular thyroid carcinoma.

pT‐stage, *n* (%)	
pT1a	8 (4.2)
pT1b	25 (13.0)
pT2	105 (54.7)
pT3a	48 (25.0)
pT3b	‐
pT4a	4 (2.1)
pT4b	‐
Missing values	2 (1.0)
pN‐stage (*n*%)	
pN0	33 (17.2)
pN1a	1 (<1)
pN1b	1 (<1)
Nx	157 (81.8)
M1, *n* (%)	3 (1.5)
Skeletal metastases	2 (1.0)
Cerebral metastases	1 (<1)
Cancer stage, *n* (%)	
Stage I	189 (98.4)
Stage II	3 (1.6)
Multifocality, n (%)	3 (1.6)
Angioinvasion, n (%)	118 (61.5)
Missing values	2 (1.0)
Largest diameter primary tumor, median in mm (IQR)	30.0 (24.0–45.0)
Missing values	41 (21.4%)
Minimal invasive	183 (95.3)
Widely invasive	9 (4.7)

*Note*: There were no additional missing variables beyond those specified in the table.

Abbreviations: AYA's, adolescents and young adults; IQR, interquartile range; *n*, number of patients.

In total, 183 patients (95.3%) were diagnosed with MI‐FTC, while nine patients (4.7%) were found to have WI FTC. Multifocality of the tumor was present in three patients (1.6%). Angioinvasion was found in 118 patients (61.5%). The median diameter of the primary tumor was 30 mm (IQR 24.0–45.0).

### 3.4 Treatment modalities

Treatment modalities are shown in Table 3. Surgery was performed in all patients. The majority of patients (144; 75%) underwent a two‐staged total thyroidectomy. Direct total thyroidectomy was performed in 34 patients (17.7%). Fourteen patients (7.3%) received a hemithyroidectomy as definite surgery. In total, nine patients (4.7%) had an incomplete resection based on the pathology report. Central lymph node dissection was performed in three patients (1.6%). One patient (<1%) underwent central and lateral lymph node dissection. In total, 157 patients (81.8%) received RAI.

**TABLE 3:** Treatment modalities of 192 AYA's with follicular thyroid carcinoma.

Surgery, *n* (%)	192 (100)
Hemithyroidectomy as only surgery, *n* (%)	14 (7.3)
Total thyroidectomy in one stage, *n* (%)	34 (17.7)
Total thyroidectomy in two stages, *n* (%)	144 (75.0)
Incomplete resection, *n* (%)	9 (4.7)
Central lymph node dissection, *n* (%)	3 (1.6)
Central + lateral lymph node dissection, *n* (%)	1 (<1)
Radioiodine ablation, *n* (%)	157 (81.8)

*Note*: There were no missing variables.

Abbreviations: AYA's, adolescents and young adults; *n*, number of patients.

## 4 DISCUSSION

In this large nationwide study, we report the long‐term recurrence and survival rate of AYAs with FTC in the Netherlands, and attempted to determine risk factors for recurrence. Furthermore, we described the tumor characteristics and treatment modalities within this patient group. Our study shows that AYAs with FTC have a very low recurrence risk and a very high overall survival rate, with current treatment protocols. Specifically, only 1.6% of the patients developed a recurrence and 2.6% died from any cause, during a median follow‐up of 12 years. Risk factors for recurrence could not be studied, due to the limited number of recurrences in this cohort. Moreover, FTC in AYAs is predominantly of the minimally invasive type (95%) and has a low occurrence of synchronous metastases (2.6%). The results of this study, in which the nationwide databases of NCR ^8^ and PALGA ^9^ were combined, add to the sparse data on the long‐term oncological outcomes of FTC in AYAs.

Previous reported recurrence rates of FTC vary widely from 2% to 29%, mainly due to differences in age distribution, inclusion of OCA, differentiation between MI‐FTC and WI‐FTC, and differences in follow‐up duration.^15^, ^16^, ^17^, ^18^, ^19^, ^20^, ^21^, ^22^, ^23^ Recently, Grønlund et al. performed a systematic review on the risk factors of recurrence of FTC.^14^ They included two studies regarding pediatric FTC ^18^, ^23^ and seven studies with patients of all ages,^15^, ^16^, ^17^, ^19^, ^20^, ^21^, ^22^ totaling 1544 patients, and found an overall recurrence rate of 14%. Excluding the two studies on pediatric FTC ,^18^, ^23^ the mean age of the remaining studies ranged from 43 to 58 years,^15^, ^16^, ^17^, ^19^, ^20^, ^21^, ^22^ which is substantially higher than in our AYA cohort. This may be a contributing factor to our low recurrence and high survival rates, considering that a higher age is a risk factor for both recurrence and mortality in FTC.^14^, ^24^, ^25^ The results of our study confirm that AYAs with FTC have a low risk for recurrence and mortality. Notably, the three patients that developed a recurrence were at the higher end of the AYA age range, specifically 38 and 39 years old.

The remarkably favorable prognosis in AYAs might also be explained by the fact that 95% of the patients had MI‐FTC. MI‐FTC is associated with low recurrence and survival rates comparable to those of the general population, in contrast to WI‐FTC.^26^, ^27^ In previous studies, the percentage of MI‐FTC in non‐pediatric cohorts ranged between 22% and 85%,^27^ a percentage considerably lower than that observed in our AYA cohort. Despite the favorable prognosis, recurrences and death do occur in a low percentage of MI‐FTC patients,^28^ which is also reflected in our data.

Only two patients (1.0%) presented with cervical lymph node metastases in our cohort. This is slightly lower compared to previous studies in adult patients with FTC, showing rates between 3% and 9% of lymph node metastases at presentation.^29^, ^30^, ^31^ Similarly, the percentage of AYAs that presented with distant metastases (three out of 192 patients; 1.5%) is remarkably lower than in previous studies on adult FTC, which reported rates between 8% and 22% for distant metastases at presentation.^29^, ^30^, ^31^, ^32^ The low occurrence of synchronous metastases in AYAs may be attributed to the fact that the median age within our cohort is lower at 31 years, in contrast to previous studies on adult FTC with a mean age between 46 and 65 years.^29^, ^30^, ^31^, ^32^ Given the well‐established association between older age and the increased risk of presenting with synchronous metastases,^24^, ^33^ the young median age of our AYA cohort likely contributes to the observed lower rate of metastatic occurrences. Again, the low rate of synchronous metastases might also be attributed to the high rate of MI‐FTC in our AYA cohort (95%), as this tumor type is associated with low rates of locoregional and distant metastases at presentation.^27^

Substantial controversy exists about the extent of treatment necessary for MI‐FTC patients. In our cohort, all patients underwent surgery, with 93% undergoing a total thyroidectomy (either in one or two stages). In total, 82% of the patients received RAI. During this period (2000–2016), the Dutch national guidelines recommended treating all MI‐FTC with total thyroidectomy and RAI. A lobectomy might be considered, in consultation with the patient, for MI‐FTC tumors smaller than 4 cm without angioinvasion and cN0.^34^ However, current international guidelines generally recommend a lobectomy for MI‐FTC smaller than 4 cm and clinically node‐negative (cN0) status.^35^, ^36^ The more aggressive treatment regime recommended by the Dutch national guidelines might also partially explain why the recurrence rate in our cohort is lower than in other studies.^15^, ^16^, ^17^, ^18^, ^19^, ^20^, ^21^, ^22^, ^23^

Goffredo et al. retrospectively analyzed 1200 MI‐FTC patients and showed no improvement in the survival of patients that underwent total thyroidectomy compared to those that underwent partial thyroidectomy, during a follow‐up of 10 years. In addition, RAI therapy had no impact on survival.^26^ Other authors reported similar conclusions for patients <45 years of age.^28^, ^37^ Consequently, it is conceivable that a portion of our AYA cohort might have received more aggressive treatment than strictly necessary.

There are some limitations to this study. Firstly, thyroglobulin (Tg) levels and Iodine scans were not captured in this database. Therefore, it is possible that we missed patients that presented with biochemical recurrences or distant recurrences, which are not always confirmed by histopathology. In that case, the recurrence rate of 1.6% would be an underestimation. We could not formally differentiate between recurrence and persistent disease, as we had no Tg‐values or Iodine scans to define a period of no evidence of disease. However, it is expected that any residual disease is treated within 12 months of diagnosis. Secondly, cause of death was not captured in this database. Therefore, we cannot make a statement on the disease‐specific survival of FTC in AYAs. However, we anticipate this to be very high, as only 2.6% died to any cause over a median follow‐up of 12 years. Thirdly, the pathological diagnosis of MI‐FTC is challenging, even for experienced pathologists. Since the pathological diagnoses in this national study are based on assessments from multiple pathological institutions, there may be heterogeneity in the pathological diagnoses. Lastly, this study comprises a historical cohort from 2000 to 2016 during which the Dutch national guidelines recommended a total thyroidectomy and radioiodine ablation for all patients with minimal invasive FTC (MI‐FTC).^34^ Since the publication of the American Thyroid Association guidelines for adult patients with thyroid nodules and DTC in January 2016,^36^ the common practice in the Netherlands has been to perform a lobectomy in patients with MI‐FTC <4 cm and cN0. Consequently, this group represents only a small part of our cohort. The current treatment practices could impact the long‐term oncological outcomes.

In conclusion, the results of this nationwide study suggest that FTC in AYAs is generally characterized as a low‐risk tumor, as it exhibits a very low recurrence rate, a high overall survival rate, and it typically presents as MI‐FTC without synchronous metastases. In this study, no risk factors for recurrence could be determined, due to the low number of recurrences. These findings should be taken into consideration when providing counseling to AYAs concerning their long‐term oncological prognosis, particularly emphasizing on the favorable prognosis associated with MI‐FTC in the absence of synchronous metastases. More research should be conducted within this specific age group, focusing on identifying high‐risk individuals for recurrence and disease‐specific mortality. Conducting these studies could lead to a more personalized treatment for AYAs with FTC, thereby improving the oncological outcomes for our high‐risk patients, while simultaneously mitigating the risk of overtreatment for those at lower risk.

## AUTHOR CONTRIBUTIONS

**Daniël J. van de Berg**: Conceptualization; data curation; formal analysis; investigation; methodology; project administration; writing—original draft; writing—review & editing. **Christiaan F. Mooij**: Supervision; Writing—original draft; writing—review & editing. **A. S. Paul van Trotsenburg**: Supervision; writing—review & editing. **Hanneke M. van Santen**: Supervision; writing—review & editing. **Sheila C. E. J. Terwisscha van Scheltinga**: Supervision; writing—review & editing. **Menno R. Vriens**: Funding acquisition; supervision; writing—original draft. **Schelto Kruijff**: Funding acquisition; supervision; writing—review & editing. **Els J. M. Nieveen van Dijkum**: Supervision; writing—review & editing. **Anton F. Engelsman**: Conceptualization; funding acquisition; methodology; supervision; writing—review & editing. **Joep P. M. Derikx**: Conceptualization; funding acquisition; methodology; supervision; writing—review & editing.

## CONFLICT OF INTEREST STATEMENT

All authors declare that they have no conflicts of interest.

## ETHICS STATEMENT

This study is approved by the institutional review board of Amsterdam University Medical Centers (registration number #20.319).

## Data Availability

The data that support the findings of this study are available from “the Netherlands Comprehensive Cancer Organization (IKNL)” and “Pathology databanking and biobanking in The Netherlands (PALGA)”. Restrictions apply to the availability of these data, which were used under license for this study. Data are available at Home (iknl.nl) or Impact door inzicht | Palga with the permission of IKNL/PALGA.
